# Characterizing the Solvated Structure of Photoexcited [Os(terpy)_2_]^2+^ with X-ray Transient Absorption Spectroscopy and DFT Calculations

**DOI:** 10.3390/molecules21020235

**Published:** 2016-02-19

**Authors:** Xiaoyi Zhang, Mátyás Pápai, Klaus B. Møller, Jianxin Zhang, Sophie E. Canton

**Affiliations:** 1 X-ray Sciences Division, Argonne National Laboratory, 9700 South Cass Avenue, Argonne, IL 60439, USA; 2 Department of Chemistry, Technical University of Denmark, Kongens Lyngby DK-2800, Denmark; papai@kemi.dtu.dk (M.P.); klaus.moller@kemi.dtu.dk (K.B.M.); 3 Wigner Research Centre for Physics, Hungarian Academy of Sciences, “Lendület” (Momentum) Femtosecond Spectroscopy Research Group, P.O. Box 49, Budapest H-1525, Hungary; 4 School of Environmental and Chemical Engineering, Tianjin Polytechnic University, Tianjin 300387, China; zjx1980@126.com; 5 Deutsches Elecktronen Synchrotron (DESY), Notkestr. 85, Hamburg 22607, Germany; secanton2012@gmail.com; 6 IFG Structural Dynamics of (Bio)Chemical Systems, Max Planck Institute for Biophysical Chemistry, Am Fassberg 11, Goettingen D-37077, Germany

**Keywords:** X-ray transient absorption spectroscopy, excited-state, osmium polypyridyl complex

## Abstract

Characterizing the geometric and electronic structures of individual photoexcited dye molecules in solution is an important step towards understanding the interfacial properties of photo-active electrodes. The broad family of “red sensitizers” based on osmium(II) polypyridyl compounds often undergoes small photo-induced structural changes which are challenging to characterize. In this work, X-ray transient absorption spectroscopy with picosecond temporal resolution is employed to determine the geometric and electronic structures of the photoexcited triplet state of [Os(terpy)_2_]^2+^ (terpy: 2,2′:6′,2″-terpyridine) solvated in methanol. From the EXAFS analysis, the structural changes can be characterized by a slight overall expansion of the first coordination shell [OsN_6_]. DFT calculations supports the XTA results. They also provide additional information about the nature of the molecular orbitals that contribute to the optical spectrum (with TD-DFT) and the near-edge region of the X-ray spectra.

## 1. Introduction

Optimizing the photochemical conversion of solar energy into useable electrochemical potential has become an important cross-disciplinary research area where the close collaboration between experiment and theory has enabled rapid progress with definite impact on device performance. Since their first implementation in 1991 [1], third generation solar cells assembled from dye-sensitized electrodes of nanocrystalline semiconductor materials have been continuously investigated as promising photovoltaic (PV) alternatives for clean energy production [1,2,3]. These relatively low-cost dye-sensitized solar cells (DSSC) can operate under low illumination and at relatively elevated temperatures. The rational design of novel interfaces demonstrating improved efficiency relies on understanding the photo-conversion process down to the atomic level.

The light-harvesting capabilities and the electronic band edge alignment underpinning the functionality of the DSSCs are largely governed by the properties of the dye moiety. Molecular complexes built around transition metal ions remain the chromophores of choice for hybrid organic-inorganic DSSCs. They combine excellent thermal and redox stability with unique photophysical attributes, such as very high optical absorption coefficients and long-lived emission. Additionally, the characteristics of their excited-state manifolds can be finely tuned through versatile substitutions within their ligand system.

Specifically, osmium (Os) molecular complexes, which belong to the so-called “red sensitizers” family, can be chemically-modified in order to realize panchromatic absorption. As a result of prominent relativistic effects in this high-Z element (Z = 76), the impact of the spin-orbit interaction cannot be neglected: the metal-to-ligand charge transfer (MLCT) bands acquire mixed singlet-triplet character [4]. Unlike for most of the ruthenium-based analogues, the absorbance now covers the full visible range ~400 nm to 800 nm, thereby matching a larger fraction of the solar spectrum. DSSCs incorporating various Os molecular complexes have indeed achieved high incident photon-to-electron conversion efficiency deep into the near infrared region [5,6,7,8,9,10,11]. Concurrently, the strong spin-orbit coupling (SOC) increases the d-d splitting, moving the positions of the metal-centered states higher up in energy when compared to their Ru analogues, thereby improving the thermal stability of the photoexcited complexes. In addition, this strong SOC influences the rate of intersystem crossing in the free chromophores, as well as the rates of “hot” (from the nominally ^1^MLCT) and “thermalized” (nominally ^3^MLCT) electron injection into the semiconductor conduction band, when the chromophores are adsorbed on the electrodes. These distinct advantages are clear motivations for experimental and theoretical investigations of their ultrafast structural dynamics. Until recently, such information was only indirectly available from low-temperature spectroscopy or transient optical absorption spectroscopy measurements.

The implementation of X-ray transient absorption (XTA) spectroscopy at storage ring facilities has opened up for the possibility to track on the 100 ps time scale the correlated changes in geometric and electronic structure of metastable species with lifetime as short as 100 ps [12,13]. Being an element specific probe of the local environment in any physical phase, it provides unique diagnostic about the correlated changes in oxidation state, coordination geometry around the absorbing atom. This is particularly well adapted to the characterization of sensitizers. Previous work has focused on [Ru(bpy)_3_]^2+^ [14,15,16] and [Ru(dcbpy)_2_(NCS)_2_]^2+^ (dcbpy: 4,4′-dicarboxy-2,2′-bipyridine)on TiO_2_ [13]. Applying the technique to the challenging case of Os complexes has necessitated drastic improvements on the setups, such as liquid-jet system reliability and long-term X-ray beam stability. The increase in signal to noise ratio has recently enabled the acquisition of XTA traces of a quality approaching that of static spectra. It has been possible to achieve an accuracy of ~0.01 Å in the determination of the excited state (ES) structure of [Os(bpy)_2_dcbpy]^2+^ for a sub-mMol concentration [17]. In this work, we report XTA spectroscopy measurements on the photoexcited triplet state of [Os(terpy)_2_]^2+^ (terpy: 2,2′:6′,2″-terpyridine) solvated in methanol. The interpretation of the results is supported by DFT and TD-DFT calculations that take into account relativistic effects (ZORA).

## 2. Results and Discussion

### 2.1. Transient XANES and EXAFS

**Figure 1:**
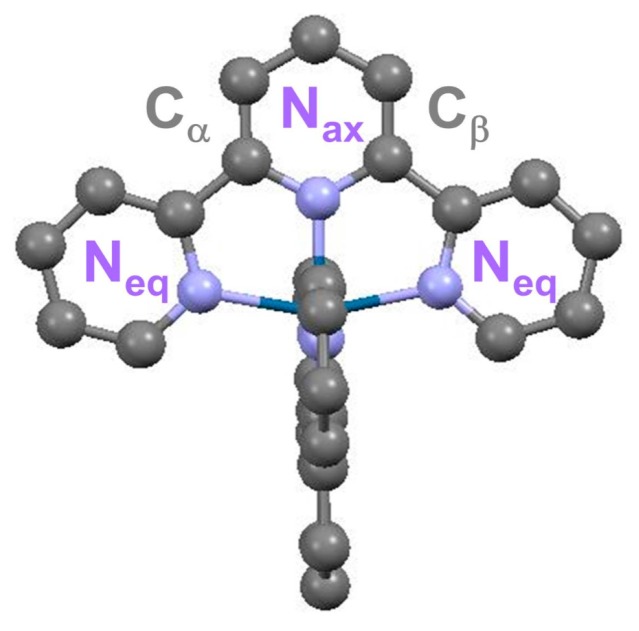
Molecular structure of [Os(terpy)_2_]^2+^.

The structure of the [Os(terpy)_2_]^2+^ in the ^1^A_1_ ground state (GS) is given in Figure 1, where the labels used in the fitting model of the XA spectra are also indicated. The tridentate coordination of the two terpyridine ligands imposes some degree of distortion in the first coordination sphere of the Os^II^ center, with two axial and four equatorial inequivalent Os-N bond lengths, denoted Os-N_ax_ and Os-N_eq_, respectively.

**Figure 2:**
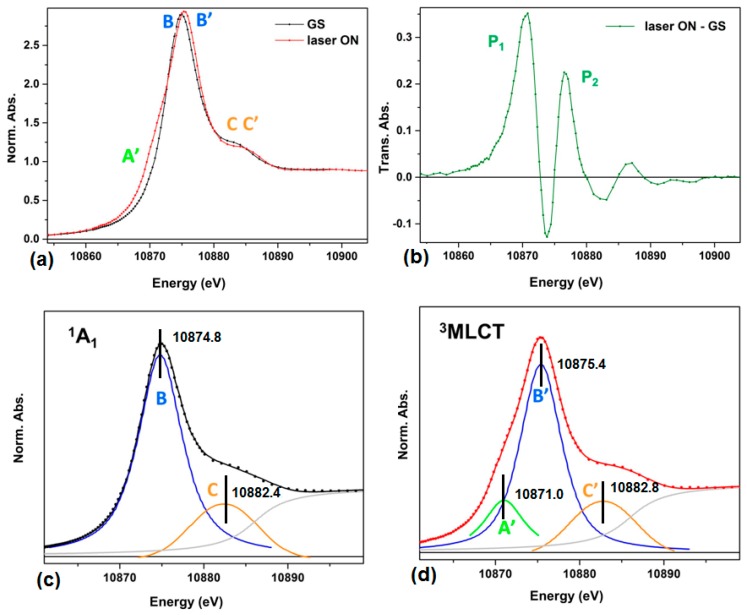
(**a**) Os L_III_ edge XANES spectra of a 1.2 mM solution of [Os(terpy)_2_]^2+^ in methanol without laser excitation (black) and with laser excitation (red) at 500 ps delay; (**b**) transient XANES spectrum produced between laser_ON and GS in (**a**); XANES spectra (solid circles) of [Os(terpy)_2_]^2+^ solvated in methanol in (**c**) the ^1^A_1_ ground state and in the (**d**) ^3^MLCT excited state. The solid lines are the results of the fit to the model described in the main text. The individual contributions from the edge jump and the participating absorption bands also displayed.

The normalized X-ray absorption near edge fine structure (XANES) spectrum of GS [Os(terpy)_2_]^2+^ in MeOH (at 1.2 mM concentration) is shown in Figure 2a (black trace). Upon photoexcitation at 527 nm, a fraction of the molecules initially in the ^1^A_1_ GS is promoted to the singlet metal to ligand charge transfer (^1^MLCT) state, and relax rapidly to ^3^MLCT state on a timescale of sub-ps to tens of ps specific for Os^II^ complexes [18,19,20].

This excited state (ES) exhibits a lifetime around 200 ns in aerated solution at room temperature [21]. Taking the difference between the laser_ON and the laser_OFF (GS) traces gives the transient XANES spectrum shown in Figure 2b.

The red trace in Figure 2a is the X-ray absorption near edge fine structure (XANES) spectrum acquired for a laser pump-X-ray probe delay of 500 ps. The intense band (the so-called “white line”) is assigned to the dipole allowed transition from the occupied 2p_3/2_ atomic level to the lowest molecular orbital with appreciable unoccupied density of states localized around the Os^II^ center. As such, the L_III_-edge spectrum indirectly reflects the extent of the 5d population in Os^II^, possibly affected by further hybridization. According to ligand field theory, the degenerate 5d levels split into t_2g_ and e_g_ levels under the influence of the quasi-octahedral field created by the two coordinated terpyridine ligands. The six electrons of Os^II^ entirely fill the t_2g_ manifold. Therefore, the white line in the ^1^A_1_ can be solely ascribed to the 2p_3/2_ → e_g_ transition (feature B). On the other hand, the XANES region of the laser_ON spectrum displays an additional band (A’) at energy lower than the main band B’. This is the spectral signature of a 2p_3/2_ → t_2g_ transition which corresponds to the positive feature P1 in Figure 2b. The appearance of this feature is explained by considering the nature of the photoexcitation.

The ^3^MLCT state fraction measured by X-ray pulse at 500 ps delay is determined to be 75% by comparing the difference absorption spectrum between laser_ON and laser_OFF with the different absorption spectrum between [Os(bpy)_3_]^3+^ and [Os(bpy)_3_]^2+^. Please see [17] for a detailed description of this approach. The Os L_III_-edge XANES spectra of the ground and the ^3^MLCT excited state are fitted with a sum of an arctangent function, the edge jump absorption, and several *pseudo*-Voigts functions to represent the absorption bands. The peak positions extracted from the data analysis were labeled in Figure 2c,d. The transition peaks of B and B’ is 10,874.8 eV and 10,875.4 eV respectively, indicating 0.6 eV blue shift of 2p_3/2_ to e_g_ transition in the ^3^MLCT state compared to the ^1^A_1_ state. The energy difference between A’ and B’ is 4.4 eV and directly measures amplitude of 5d orbitals splitting in the ^3^MLCT state.

**Figure 3:**
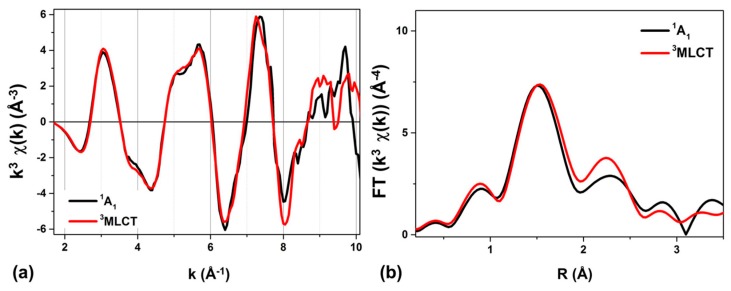

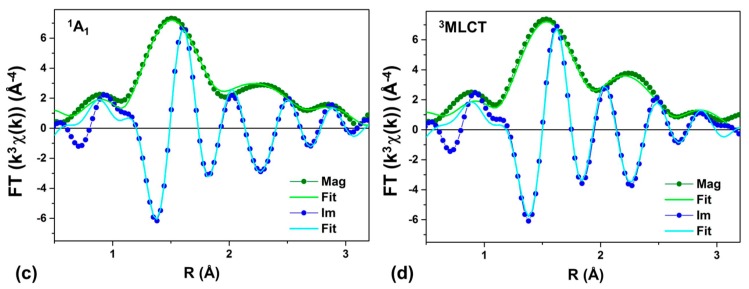
(**a**,**b**) Os L_III_—edge EXAFS oscillation, weighted by k^3^, where k is the photoelectron wavevector for the ^1^A_1_ state (black) and the ^3^MLCT excited state (red) in (**a**) k space and in (**b**) R space; (**c**,**d**) Magnitude (green dots) and imaginary (blue dots) parts of the Fourier transform of the k^3^ weighted EXAFS oscillations, along with their best fits (green and blue solid lines respectively) to the model described in the text; for (**c**) the ^1^A_1_ state and for (**d**) the ^3^MLCT excited state. It should be noted that these spectra are phase uncorrected, so that the distance R of the maximum distribution are actually smaller than the actual experimental average Os-N bond lengths for both states.

Denoting k as the photoelectron wavevector, Figure 3a displays the k^3^ weighted extended X-ray absorption fine structure (EXAFS) χ(k) in k space for the GS (black) and ES (red) states, and Figure 3b shows the corresponding magnitude of the phase-uncorrected Fourier transform (FT) of k^3^ χ(k). The peaks located within the 1–2 Å range are associated with the respective average Os-N bond distance, and only a slight shift to higher R values can be observed for the laser-excited species in the ^3^MLCT. This finding is in line with the slight structural changes expected for the family of Os^II^ polypyridyl complexes [17]. The FT of k^3^ χ(k) with 2.4 Å^−1^ < k < 10 Å^−1^ was fitted in R space restricted to the 1.1 Å–3.1 Å interval. The scattering contributions from all the paths in the first two coordination shells (Os-N and Os-C_α,β_ in Figure 1) were included in the model. The resulting fitted magnitude and imaginary parts of the FT of k^3^ χ(k) are respectively presented in Figure 3c,d for the ^1^A_1_ and ^3^MLCT state. The principal structural parameters are summarized in Table 1. The averaged Os-N_ax_ and Os-N_eq_ bond-length is (1.982 ± 0.007) Å and (2.069 ± 0.007) Å in the GS, respectively. The ^3^MLCT state shows slightly Os-N bond elongation of (0.02 ± 0.01) Å.

### 2.2. DFT-Based Calculations

**Figure 4:**
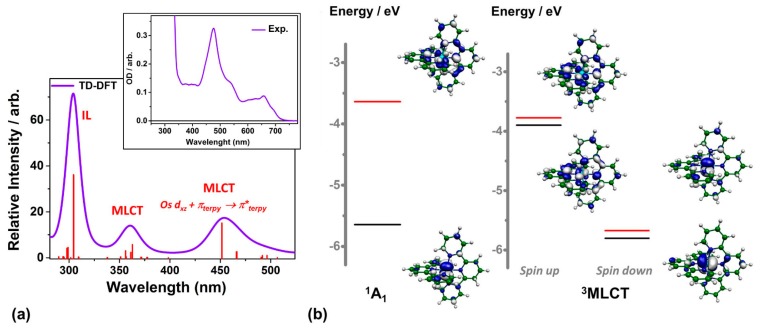
(**a**) Optical absorption spectrum simulated by TD-DFT. The main character of the transitions is indicated above the photo excited band. The experimental spectrum is shown in the inset; (**b**) The HOMO and LUMOS of the ^1^A_1_ and ^3^MLCT states obtained from the DFT optimization.

The optical spectrum simulated with TD-DFT (B3LYP and ZORA) is shown in Figure 4a. It is in good qualitative agreement with the experimental spectrum shown in the inset and the ones reported in the literature [22]. The intense absorption band centered around 304 nm originates from interligand (IL) transitions and the other two transient bands centered at 360 nm and 453 nm correspond to transitions with mainly MLCT character. The HOMO-LUMO of the singlet GS and triplet ES from the calculations are displayed in Figure 4b. The HOMO of the ^1^A_1_ state possesses mainly non-bonding Os 5d character while its LUMO has substantial contributions from orbitals located on the two terpyridine ligands. Such results clearly show that the ES corresponds to electron transfer from Os 5d to ligands. The HOMO of GS becomes the LUMO of ES, giving an extra 2p–5d transition feature in the XANES spectrum of ES. (Figure 2) According to the DFT calculations, the ES lies 1.94 eV above the GS. The ^3^MC is much higher at 3.9 eV as obtained from the TD-DFT calculation performed at the Franck-Condon (FC) geometry. Both factors are favorable for obtaining the long ES lifetimes necessary for PV applications.

The DFT optimization of the molecular complex solvated in methanol (MeOH) (using B3LYP and ZORA) establishes that Os-N_ax_ = 1.993 Å and Os-N_eq_ = 2.074 Å with a bite angle N_eq_-Os-N_ax_ of 78.6°. These values are in excellent agreement with the reported crystal structure of [Os(terpy)_2_][ClO_4_]_2_·(H_2_O)_0.5_ [23,24] and previous DFT calculations for [Os(terpy)_2_]^2+^ in acetonitrile [18]. The Os-N_ax_ and Os-N_eq_ bond-lengths also agrees well with those extracted from XTA analysis (Table 1). The structure of the triplet ES has also been optimized with the DFT method. The results show that the change in Os-N bond lengths are negligible, Os-N_ax_ by +0.006 Å and a bond contraction of Os-N_eq_ by −0.004 Å. Based on the calculation, the bite angle N_eq_-Os-N_ax_ opens from 78.6° to 79.1° (*i.e.*, +0.5°) and the terpyridine ligand, which was previously quasi-planar in the GS, now becomes ruffled. Calculations with the BP86 functional and pseudo potential on Os^II^ (instead of ZORA) return similar results.

## 3. Experimental Section

### 3.1. X-ray Transient Absorption (XTA) Measurement

The XTA measurements were carried out at 11-ID-D of the Advanced Photon Source (APS) at Argonne National Laboratory (Argonne, IL, USA). The laser pump pulse was the second harmonic output of a Nd:YLF regenerative amplified laser at 527 nm, 1.6 kHz repetition rate, 5 ps FWHM (full width half maximum). The experiment was carried out under standard operation mode. The intense X-ray pulse with 78 ps FWHM and 6.5 MHz repetition rate was used as the probe. [Os(terpy)_2_]^2+^ dissolved in methanol (1.2 mM) was flowed through a stainless steel tube and formed a free jet of 550 µm in diameter. Two avalanche photodiodes (APDs) positioned at 90° angle on both sides of the incident X-ray beam collected the X-ray fluorescence signals. A soller slits/Zn filter combination, which was custom-designed for the specific sample chamber configuration and the distance between the sample and the detector, was inserted between the sample fluid jet and the APD detectors.

The outputs of the APDs were sent to two fast analyzer cards (Agilent, Santa Clara, CA, USA) that were triggered by TTL signal synchronized with laser pulses. The card digitized the X-ray fluorescence signals as a function of time at 1 ns/point after each trigger and averaged repeated measurements at a 4s integration time. The fluorescence from the synchronized X-ray pulse at 500 ps after the laser excitation was used for building the ES spectrum and the GS spectrum was obtained by averaging the intense X-ray pulses in the previous 50 synchrotron ring cycles.

### 3.2. XANES Data Analysis

The Os L_III_-edge XANES spectra of the ground and the ^3^MLCT ES were fitted with a sum of an arctangent function for the edge jump absorption, and several pseudo-Voigt functions to represent the transition bands. The ionization potential is fixed at 10,884.0 eV and 10,886.2 eV for the GS and ^3^MLCT respectively based on the EXAFS data analysis.

### 3.3. EXAFS Data Analysis

The Athena program is used to process experimental XAS data to extract the normalized oscillation amplitude χ^exp^(*k*) and the photoelectron wave number *k* is defined by k=2m(E−E0)ħ, where *E_0_* is the absorption edge energy. The theoretical calculated *χ^th^(k)* is given by EXAFS equation:

$$\chi^{th}\left(k\right)=\underset{j}{\sum }\frac{S_{0}^{2}N_{j}f_{j}\left(k\right)}{kR_{j}^{2}}e^{-2k^{2}\sigma_{j}^{2}}e^{-2r_{j}/\lambda \left(k\right)}\sin\left[2kR_{j}+\delta_{j}\left(k,r_{j}\right)\right]$$

where *j* indicates a shell with identical backscatters, N*_j_* is the coordination number of *j*th shell, *f_j_* is the backscattering amplitude, *R_j_* is the average distance, σ*_j_* is the mean square variation, δ*_j_* is the scattering phase shift, λ is the effective mean free path and ${\text{S}}_{0}^{2}$ is the amplitude reduction factor.

FEFF6 is used to calculate *f_j_*, δ*_j_* and λ. Fitting to the experimental data to refine the structure parameters ${\text{S}}_{0}^{2}$, *N_j_*, *R_j_*, $\sigma_{\text{j}}^{2}$ is done using the Artemis program. Crystal structure of [Os(terpy)_2_]^2+^ from X-ray diffraction (XRD) measurement (CSD code:GOGDOV) [24] is used as the starting structure for fitting of both ^1^A_1_ and ^3^MLCT states. The contributions from all the paths in the first two scattering shells Os-N, Os-C, were included in the fitting. Each shell shares a common $\sigma_{j}^{2}$ and distance changes from the input structure. All paths share a common $S_{0}^{2}$ and *E*_0_.

### 3.4. DFT Calculations

The lowest-lying singlet (^1^A_1_) and triplet (^3^MLCT) electronic states of [Os(terpy)_2_]^2+^ were fully optimized with the application of the gradient corrected BP86 [25,26] exchange-correlation functional in conjunction with the scalar relativistic (ZORA) TZVP atomic basis set [27] as implemented in the ORCA3.0 program package [28]. Solvation effects were taken into account by using the conductor-like screening model (COSMO) [29] with the selection of the dielectric constant of methanol (ε = 32.6). Frontier Kohn-Sham orbitals were extracted from the converged wave function. In order to obtain accurate electronic energies of the GS and ^3^MLCT states, the DFT optimizations were repeated using the B3LYP* [30]/ZORA-TZVP method, which provided accurate spin-state energetics for related transition metal complexes [31,32].

## 4. Conclusions

We have obtained high-resolution XANES and EXAFS spectra of solvated [Os(terpy)_2_]^2+^ in methanol in both the GS and the ^3^MLCT photo-excited state. The XANES revealed important information regarding orbital energy levels. The 2p_3/2_ → e_g_ transition in the ^3^MLCT photo-excited state blue shifts 0.6 eV compared to GS. The energy difference between two spitted 5d orbital levels is 4.4 eV. The EXAFS spectra allow us to resolve an average Os-N bond distance change of (0.02 ± 0.01) Å in the ES. The Os-N bond-lengths obtained from DFT calculations agree with the experimental results. The calculations provide additional information about the nature of the molecular orbitals that contribute to the optical spectrum (with TD-DFT) and the near-edge region of the X-ray spectra.

## Figures and Tables

**Table 1:** Electronic and structural parameters of [Os(terpy)_2_]^2+^ in ground and ^3^MLCT states solvated in methanol, as determined by XTA and DFT calculations. The values that were kept fixed during the fitting procedure are indicated in italic.

Method	Bond	^1^A_1_			^3^MLCT				
		(E_0_ = 10884.0 ± 1.4 eV, S_0_^2^ = 1)			(E_0_ = 10886.2 ± 1.3 eV, S_0_^2^ = 1)			ΔE_B’-A’_ (eV)	ΔE_B’-B_ (eV)
		N	R(Å)	σ^2^(Å^2^)	N	R(Å)	σ^2^(Å^2^)	4.40 ± 0.07	0.60 ± 0.04
XTA	Os-N_ax_	2	1.982 ± 0.007	0.003 ± 0.001	6	2.002 ± 0.007	0.003		
	Os-N_eq_	4	2.069 ± 0.007	0.003 ± 0.001		2.089± 0.007	0.003		
DFT	Os-N_ax_	2	1.993		2	1.999			
	Os-N_eq_	4	2.074		4	2.070			

## Abbreviations

The following abbreviations are used in this manuscript:

XTA: X-ray transient absorption
DSSC: dye-sensitized solar cells
MLCT: metal-to-ligand charge transfer
SOC: spin-orbit coupling
GS: ground state
ES: excited state
XANES: X-ray absorption near edge fine structure
EXAFS: extended X-ray absorption fine structure
FC: Franck-Condon
LD: linear dichroism
